# Green tea extract supplement reduces D-galactosamine-induced acute liver injury by inhibition of apoptotic and proinflammatory signaling

**DOI:** 10.1186/1423-0127-16-35

**Published:** 2009-03-25

**Authors:** Bor-Ru Lin, Chia-Jung Yu, Wang-Chuan Chen, Hsuan-Shu Lee, Huei-Min Chang, Yen-Chih Lee, Chiang-Ting Chien, Chau-Fong Chen

**Affiliations:** 1 Graduate Institute of Physiology, National Taiwan University College of Medicine, Taipei, Taiwan; 2 Department of Integrated Diagnostics and Therapeutics, National Taiwan University Hospital, Taipei, Taiwan; 3 Department of Internal Medicine, National Taiwan University Hospital and National Taiwan University College of Medicine, Taipei, Taiwan; 4 Department of Biochemistry, Chang-Gung University, Taoyuan, Taiwan; 5 Departments of Infectious Disease Control and Clinical Immunology and Immunology and Microbiology, Nihon University School of Medicine, Tokyo, Japan; 6 Division of Chinese Medicine, Ren-Ai Branch, Taipei City Hospital, Taipei, Taiwan; 7 Departments of Medical Research, National Taiwan University Hospital and National Taiwan University College of Medicine, Taipei, Taiwan; 8 Department of Surgery, Kuan-Tien General Hospital, Taichung, Taiwan

## Abstract

Oxidative stress and inflammation contributed to the propagation of acute liver injury (ALI). The present study was undertaken to determine whether D-galactosamine (D-GalN) induces ALI via the mitochondrial apoptosis- and proinflammatory cytokine-signaling pathways, and possible mechanism(s) by which green tea (GT) extract modulates the apoptotic and proinflammatory signaling in rat. D-GalN induced hepatic hypoxia/hypoperfusion and triggered reactive oxygen species (ROS) production from affected hepatocytes, infiltrated leukocytes, and activated Kupffer cells. D-GalN evoked cytosolic Bax and mitochondrial cytochrome C translocation and activated proinflammatory nuclear factor-kappa B (NF-κB) and activator protein-1 (AP-1) translocation, contributing to the increase of intercellular adhesion molecule-1 expression, terminal deoxynucleotidyl transferase-mediated nick-end labeling (TUNEL)-positive hepatocytes, multiple plasma cytokines and chemokines release, and alanine aminotransferase (ALT) activity. An altered biliary secretion profile of several acute phase proteins directly indicates oxidative stress affecting intracellular trafficking in the hepatocyte. GT pretreatment attenuated ROS production, mitochondrial apoptosis- and proinflammatory cytokine-signaling pathway, plasma ALT and cytokines levels, biliary acute phase proteins secretion and hepatic pathology by the enhancement of anti-apoptotic mechanisms. In conclusion, D-GalN induced ALI via hypoxia/hypoperfusion-enhanced mitochondrial apoptosis- and proinflammatory cytokine-signaling pathway, contributing to oxidative stress and inflammation in the liver. GT can counteract the D-GalN-induced ALI via the attenuation of apoptotic and proinflammatory signaling by the upregulation of anti-apoptotic mechanism.

## Background

Acute liver injury (ALI) may cause dismal clinical outcome [1,2], but the detailed pathophysiologic mechanisms and the preventive and therapeutic medications have not been fully elucidated. In all types of liver damage there is consistent evidence of enhanced oxidative stress and/or significant decrease of antioxidant defense [2]. Oxidative stress and inflammation have been reported to contribute to the pathogenesis of alcohol-, CCl_4_-, thioacetamide- and endotoxin-induced ALI [2-6]. D-galactosamine (D-GalN) treated livers have metabolic and morphological aberrations similar to those observed in human viral hepatitis that always caused peri-portal necro-inflammation [7,8] and hepatocyte apoptosis [9]. Although D-GalN was well-known to induce toxicity by blocking RNA and protein synthesis [7,8], its ability to induce oxidative injury in the liver was still poorly delineated.

Reactive oxygen species (ROS) play a crucial role in the induction and in the progression of liver disease. In response to hypoxia/hypoperfusion or toxic injury, a massive ROS production can cause lipid peroxidation of cellular membranes, and protein and DNA oxidation, which results in cellular injury [2,10-14]. The main sources of ROS may derive from the mitochondria of hepatocytes, the activated macrophages (Kupffer cells), and the infiltrating neutrophils [2,11-14]. These ROS can trigger the translocation of nuclear factor-kappa B (NF-κB) and activator protein-1 (AP-1) to nucleus [12] and activation of inflammatory cytokines, chemokines, and adhesion molecules that, in turn, can contribute to further production of ROS [2,6,14] and consecutively activate the cascade of Bax and cytochrome c translocation and caspases (apoptosis) [15]. Various kinds of antioxidants capable of decreasing NF-κB activity, ameliorating mitochondrial dysfunction, cytochrome c release and caspase-3-mediated apoptosis, and decreasing inflammatory cell infiltration [2-5,15-17] have been shown to reduce tissue injury. Recently, the ROS enhanced proinflammatory NF-κB, AP-1, and intercellular adhesion molecule-1 (ICAM-1) expression as well as promoted proapoptotic mechanisms, including increases in the Bax/Bcl-2 ratio, in CPP32 expression, in poly-(ADP-ribose)-polymerase (PARP) cleavages, and in DNA fragmentation and apoptotic cells in the liver can be inhibited by the GT extract supplement [12]. The cotreatment of epigallo-catechin gallate resulted in the complete protection of the hepatocyte apoptosis suppressing the increases of caspase-3 in the cytoplasm [12]. In addition, GT extract supplement palliated plasma HOCl activity more effectively than vitamin C [18]. GT extract also displayed dose-response effects on palliating hemodialyis-enhanced plasma H_2_O_2 _and HOCl activities, lipid peroxidation (hosphatidylcholine hydroperoxide) production, C-reactive protein and proinflammatory cytokines expression, and restored paraoxonase 1 activity [18].

Therefore, we hypothesize that catechins, may prevent or ameliorate ALI-associated apoptosis and inflammation induced by a toxin such as D-GalN. In the present study, we proved that D-GalN could induce ALI via promoting mitochondrial apoptosis and modulating proinflammatory cytokine-signaling pathways, and such detrimental effects could be abolished by GT extract supplement.

## Methods and materials

### Animals

Female Wistar rats (200–250 g) were housed at the Experimental Animal Center, National Taiwan University. All surgical and experimental procedures were approved by the animal care and experimental protocols were in accordance with the guidelines of the National Science Council of the Republic of China (NSC 1997).

The rats were anesthetized with subcutaneous urethane (Sigma, St. Louis, MO, USA, 1.2 g/kg). D-GalN (Sigma, St. Louis, MO, USA) was injected intraperitoneally at a dose of 400 mg/kg body weight. The arterial blood pressure and bile flow were measured [12]. At the end of each experiment, the animals were sacrificed with overdose anesthetics. Bile, plasma, and liver were stored at -70°C until analyses.

### Measurement of hepatic hemodynamics

Portal venous pressure (PVP), portal venous blood flow (PVBF), and hepatic microvascular resistance were measured as described previously [12].

### Measurement of hepatic O_2 _tension and hemodynamics

We monitored hepatic liver oxygenation in response to D-GalN at the liver as previously described [12].

### *In vivo *and *in vitro *chemiluminescence recording for ROS activity

The ROS generation in response to D-GalN-induced liver injury was measured from the liver surface, bile, and whole blood by a modified chemiluminescence detection method, as described previously [11,12,15]. Briefly, the ROS generation in response to D-GalN toxicity was measured from the liver surface by intravenous infusion of a superoxide anion probe, 2-Methyl-6-(4-methoxyphenyl)-3,7-dihydroimidazo- [1,2-a]-pyrazin- 3-one-hydrochloride (MCLA) (0.2 mg/ml/h, TCI-Ace, Tokyo Kasei Kogyo Co. Ltd., Tokyo, Japan) and by the use of a Chemiluminescence Analyzing System (CLD-110, Tohoku Electronic In. Co., Sendai, Japan) [12]. The real-time displayed chemiluminescence signal was indicated as ROS level from the liver surface.

For evaluating the effects of antioxidants on oxidative injury, identical experiments were performed in five rats fed for two week with decaffeinated green tea extract (GT, 25–125 mg/kg), which is purchased from Numen Biotech Co., Ltd., (Taipei, Taiwan) [12]. The GT extract was composed of various types of catechins (328 mg/g of epigallocatechin gallate, 152 mg/g of epicatechin gallate, 148 mg/g of gallocatechin gallate, 132 mg/g of epicatechin, 108 mg/g of epigallocatechin, 104 mg/g of galloctechin, and 44 mg/g of catechin), which is analyzed by high performance liquid chromatography (HPLC). The GT extract (25 and 125 mg) was dissolved in 500 mL of deionized distilled water every day. Each rat received restricted fresh drink (100 mL/kg body weight) daily provided at 6:00 p.m. in each cage by using sealed bottles for 2 week. For consistent dosage of GT extract, the rest of tea drink in some rats was feed at 4:00 p.m. on the second day [12].

### Analysis of catechins in rat plasma by using high performance liquid chromatography

The standard samples of epigallocatechin gallate, epicatechin gallate, gallocatechin gallate, epicatechin, epigallocatechin, galloctechin, and catechin were purchased from Sigma Chemical Co. (St. Louis, MO). Oxalic acid, ethanol, NaH_2_PO_4 _were purchased from Merck. Mobile Gradient mobile phase A and B solution were from ESA Inc. (Bedford, MA, USA). A standard stock mixture of epigallocatechin gallate, epicatechin gallate, gallocatechin gallate, epicatechin, epigallocatechin, galloctechin, and catechin at 100 μg/ml was prepared in 10 mM oxalic acid solution and stored in small aliquots at -80°C until use. The stock solutions remained stable for at least 6 months. The distribution of catechins in plasma and standards was determined by a gradient HPLC-Coulometric electrode array system (ESA Inc., Bedford, MA, USA). One ml of rat plasma samples was subjected to Al_2_O_3 _solid phase extraction (ESA Inc., Bedford, MA, USA), and the resulting samples in 1 M NaH_2_PO_4 _(pH = 2.5) were applied to the HPLC with an autosampler (Model 542, ESA) and an electrochemical detector (Model 5600A, ESA). The separating conditions were as follows: column, HR-80 (C-18, 3 μm, 4.6 mm × 80 mm, ESA); column temperature, 25°C; gradient mobile phase A (containing phosphate buffer and an ion pairing agent, 45–0171, ESA) and gradient mobile phase B (containing methanol, phosphate buffer, and an ion pairing agent, ESA); flow rate, 1 mL/min. The optionally channel potential was set to 220 mV. The peak height was used to calculate the plasma concentration using an external calibration curve of spiked control plasma as described previously in our laboratory [18]. The rat plasma epigallocatechin gallate (10.1 ± 0.3 ng/mL or 13.2 ± 1.2 ng/mL), epicatechin gallate (5.1 ± 0.6 ng/mL or 6.3 ± 0.9 ng/mL), gallocatechin gallate (4.9 ± 0.7 ng/mL or 5.7 ± 0.8 ng/mL) were detected, but epicatechin, epigallocatechin, galloctechin, and catechin were not detected in the rats with 25 mg/500 mL or 125 mg/500 mL GT extract.

### Two-dimensional electrophoresis

Bile is secreted by hepatocytes, therefore, bile proteomic profile may be provided more specific information and protein markers of the hepatic responses to D-GalN-induced injury and GT effects. The collected bile was analyzed by two-dimensional electrophoresis (2-DE) gel techniques. Isoelectric focusing was performed with 13-cm immobilized pH gradient strips pH 3–10 (IPG strips, Amersham Bioscience) using the diffusion approach for a total of 33 kVh (IPGphor II, Amersham Bioscience) at 20°C. Gels were stained with a modified silver nitrate procedure [19] according to the manufacturer's instructions (Amersham Bioscience). The silver nitrate stained protein spots were excised and subjected to in-gel tryptic digestion as the previously described [20].

### Peptide analysis by mass spectrometry

Zip-Tip cleaned samples were mixed with saturated α-cyano-4-hydroxycinnamic acid solution in acetonitrile/H_2_O and spotted onto a matrix-assisted laser desorption/ionization (MALDI) sample plate. MALDI time-of-flight mass spectrometry (MALDI-TOF MS) analysis was performed on a Voyager DE-STR workstation (PerSeptive Biosystems, Framingham, MA, USA) equipped with a 337 nm nitrogen laser. The peptide mass fingerprint data were compared to those in the NCBInr protein database using the Mascot searching tool . Protein identification was performed using Peptide Search from the Mascot searching tool. Search parameters included: Database, NCBInr; taxonomy, Homo sapiens; enzyme, trypsin; peptide charge, 1+; instrument, MALDI-QUAD-TOF.

### Electrophoretic Mobility Shift Assay (EMSA)

The nuclear extracts from liver samples were obtained with a nuclear extraction kit (Panonics, Inc. Redwood City, CA, USA). Nine μg of nuclear extracts were analyzed by using a commercially Electrophoretic-Mobility Shift Assay (EMSA, Panonics, Inc.) for identifying NF-κB and AP-1 that interacts with DNA. The EMSA procedure was followed according to the manual and final results were obtained by using a chemiluminescence imaging system [12].

### Western blot analysis

Cytosolic Bax translocation to mitochondria and mitochondrial leakage of cytochrome C to cytosol are required for triggering apoptotic pathway [17]. The livers were subjected to differential centrifugation to obtain the mitochondrial and cytosolic fractions. Ten μg of protein was electrophoresed as described below. The primary antibody was a polyclonal rabbit antihuman cytochrome C and heat shock protein 60 (HSP60) goat polyclonal antibody (Santa Cruz Biotechology, Inc., Santa Cruz, CA, USA) used at 1:1000 or Bax (Chemicon, Temecula, CA, USA).

We measured the expression of intercellular adhesion molecule-1 (ICAM-1), Bax, Bcl-2, caspase 3, PARP [12], and sterol regulatory element binding protein-1 (SREBP-1) in the total protein from livers subjected to D-DalN-induced injury. We also measured the expression of transferring and haptoglobin in the bile from livers subjected to D-DalN-induced injury.

Antibodies raised against ICAM-1 (R&D systems, Inc., Minneapolis, MN, USA), Bax (Chemicon, Temecula, CA, USA), Bcl-2 (Transduction, Bluegrass-Lexington, KY, USA), the activation fragments (17 kD of cleaved product) of caspase 3 (CPP32/Yama/Apopain) (Upstate Biotechnology, Lake Placid, NY, USA), PARP (Promega, Madison, WI, USA), SREBP-1 (Santa Cruz Biotechnology, Inc., Santa Cruz, CA, USA), transferrin (Santa Cruz), haptoglobin (Abcam, Cambridge, UK) and β-actin (Sigma, St. Louis, MO, USA) were used. Proteins on SDS-PAGE gels were transferred to nitrocellulose filters and stained as described [12].

### Immunocytochemistry for oxidative stress and apoptosis

A nitroblue tetrazolium (NBT, Sigma, St. Louis, MO, USA) perfusion method was used for localizing *de novo *ROS generation in the liver [11,12,15]. The NBT-perfused liver was removed and fixed in zinc/formalin solution and processed for histologic examination of formazan deposits. The value of blue NBT deposits/total section area was counted by Adobe Photoshop 7.0.1 image software analysis.

We also performed anti-4-hydroxy-2-nonenal (4-HNE) antiserum (Alpha Diagnostic International, Inc., San Antonia, TX, USA) and terminal deoxynucleotidyl transferase-mediated nick-end labeling (TUNEL) method [12] to determine the presence and extent of apoptotic cells as evidence of oxidative stress. The tissue sections (5 μm) of the liver were prepared, deparaffinized, and stained by the methyl green, 4-HNE- and TUNEL-avidin-biotin-complex method. Twenty high-power (× 400) fields were randomly selected for each liver section, and the value of apoptotic cells/(apoptotic cells and methyl green stained cells) was counted.

For hepatic Kupffer cell (ED1) staining, the tissue sections were incubated overnight at 4°C with a mouse anti-rat antibody to ED1 (CD68, 1:200, Serotec, Sydney, NSW, Australia). A biotinylated secondary antibody (Dako, Botany, NSW, Australia) was then applied followed by streptavidin conjugated to horse peroxidase (Dako). The chromogen used was Dako Liquid diaminobenzene. Twenty high-power (× 200) fields were randomly selected for each liver section, and the value of ED1 positive cells was counted.

### Biochemical analysis

The plasma alanine aminotransferase (ALT) level was determined by use of a commercially available analytical kit (Sigma, St. Louis, MO, USA). Bile concentrations of transferrin and haptoglobin, two representative biomarkers, were selected and measured with *Rat transferrin ELISA Kit (Wako) and Rat haptoglobin ELISA Kit (Wako)*.

### Multiple cytokine antibody arrays

In response to toxicity, several inflammatory mediators such as cytokines and chemokines could be released by activated macrophages/Kupffer cells in the damaged liver or activated blood monocytes. Therefore, multiple cytokine-expression levels were simultaneously determined and identified by use of RayBio^®^rat cytokine protein arrays (RayBiotech, Inc., Norcross, GA, USA) according to the manufacturer's instructions.

### Statistical analysis

All values were expressed as mean ± standard error of the mean. Differences within groups were evaluated by paired *t*-test. One-way analysis of variance was used for examining differences among groups. Inter-group comparisons were made with Duncan's multiple-range test. A *P *value of < 0.05 was considered to indicate significance.

## Results

### Effect of D-GalN on hepatic hemodynamics

**Figure 1:**
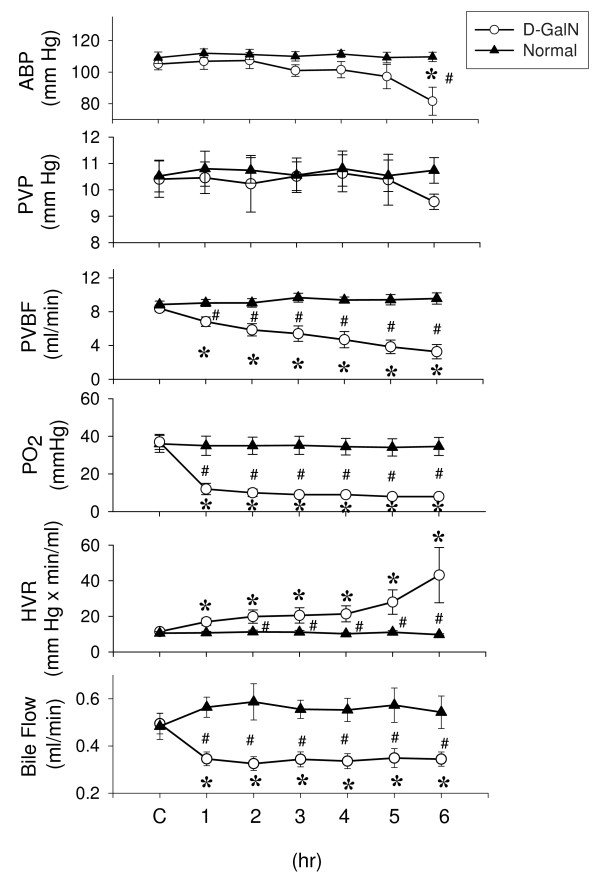
**Mean values of arterial blood pressure (ABP), portal venous pressure (PVP), portal venous blood flow (PVBF), hepatic oxygen tension (PO_2_), hepatic vascular resistance (HVR), and bile flow at different time in response to D-GalN treatment**. In D-GalN rats (n = 6), D-GalN significantly decreased the PVBF, PO_2_, and bile flow and increased HVR. In normal rats (n = 6), the hepatic vasoconstrictor effect was not found. * *P *< 0.05 vs. the control value (C). # *P *< 0.05 D-GalN *vs. *Normal.

Within 6 hr post treatment of D-GalN, a decrease in PVBF, liver oxygenation, and bile flow and an increase in hepatic vascular resistance are demonstrated (Figure 1), indicating a hypoxia/hypoperfusion condition induced by D-GalN.

### D-GalN increased ROS in liver, bile, and blood

**Figure 2:**
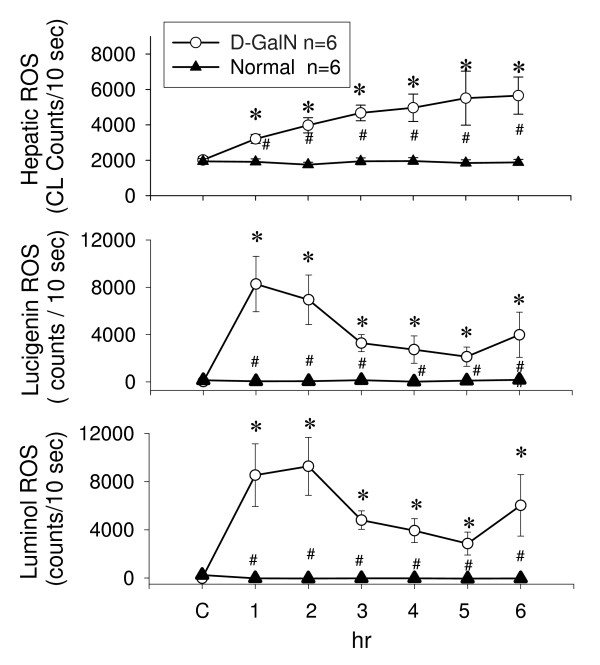
**Effects of D-GalN treatment on *in vivo *and *in vitro *ROS generation in the livers of rats**. Increase in hepatic ROS occurred in D-Galn treated rats, but not in normal rats. Mean values of hepatic ROS and bile lucigenin and luminol ROS generation measured at different times after D-GalN treatment in rats are depicted. * *P *< 0.05 vs. control value (C). # *P *< 0.05 between D-GalN and normal groups.

**Figure 3:**
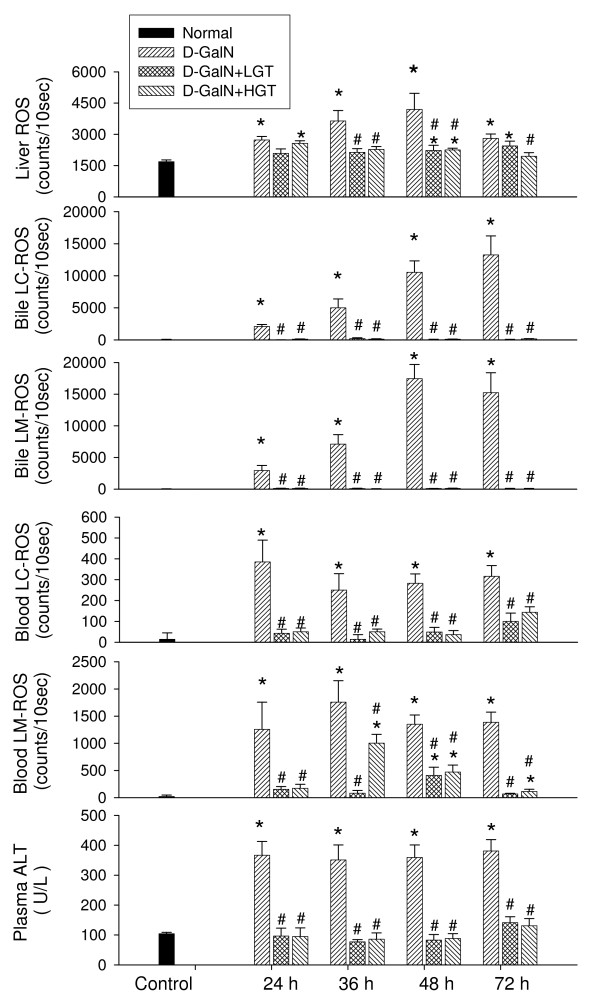
**Low dose and high dose of green tea extracts (GT) effects on liver reactive oxygen species (ROS), bile lucigenin (LC) and luminol (LM) ROS, blood LC and LM ROS, and plasma alanine aminotransferase (ALT) in D-galactosamine treated rats**. * *P *< 0.05 vs. control value (C). # *P *< 0.05 between D-GalN and GT treatment.

Intravenous infusion of MCLA measured to a baseline level of superoxide-dependent ROS counts in a range of 1500–2200 counts (Figure 2). Upon D-GalN administration, the level of O_2 _^-.^-dependent ROS from the liver surface started to increase within 6 hr and was maintained at high level at 24–72 hrs. Hepatic ROS production was accompanied by increased O_2 _^-. ^and H_2_O_2 _production in the bile within 6 hr of D-GalN treatment (Figure 2). The hepatic ROS, biliary O_2 _^-. ^and H_2_O_2_, and blood O_2 _^-. ^and H_2_O_2 _were consistently elevated at 24–72 hrs (Figure 3). However, the increased level of hepatic, biliary, and whole blood ROS was significantly reduced by the pretreatment with GT. The increased plasma ALT level was also depressed by the GT pretreatment.

### Effect of D-GalN on proinflammatory and apoptosis-regulated gene expression

**Figure 4:**
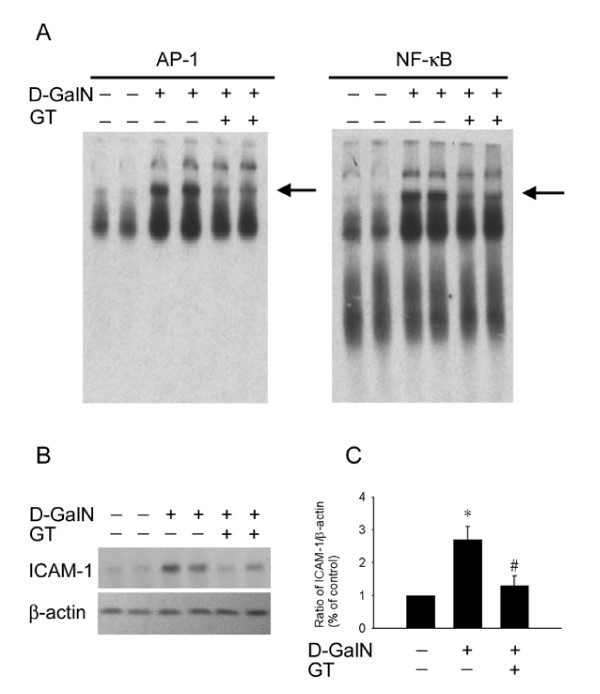
**A: The analysis of nuclear factor NF-κB and AP-1 from the nuclear extracts of liver samples at 6 hr of D-GalN treatment were performed with a nuclear extraction kit and using a commercially Electrophoretic-Mobility Shift Assay**. The induction of nuclear factor NF-κB and AP-1 nuclear factors by D-GalN treatment is partly inhibited by green tea (GT) pretreatment.**B: **Immunoblot analyses for specific antibodies to ICAM-1, and β-actin were performed in livers taken at 6 hr after D-GalN treatment. Note the increased expression of ICAM-1 in response to D-GalN. **C: **The enhanced expressions of these specific proteins were significantly reduced by GT pretreatment. Equal protein loading was displayed by β-actin. * *P *< 0.05 vs. control value. # *P *< 0.05 vs. D-GalN treatment.

ROS triggered early cellular signal transduction pathways responsible for the activation of NF-κB and AP-1, resulting in up-regulation of the ICAM-1 gene in the insulted tissue [12]. The proinflammatory response of NF-κB and AP-1 translocation and the ICAM-1 protein expression in the livers subjected to D-GalN is displayed in Figure 4. Significant hepatic nuclear binding of transcription factor NF-κB and AP-1 was observed in the D-GalN liver. The enhanced hepatic NF-κB and AP-1 binding activity was partly inhibited by GT pretreatment (Figure 4A). The ICAM-1 expression was increased by D-GalN treatment (Figures 4B &4C). The increased proinflammatory responses were inhibited by GT pretreatment.

**Figure 5:**
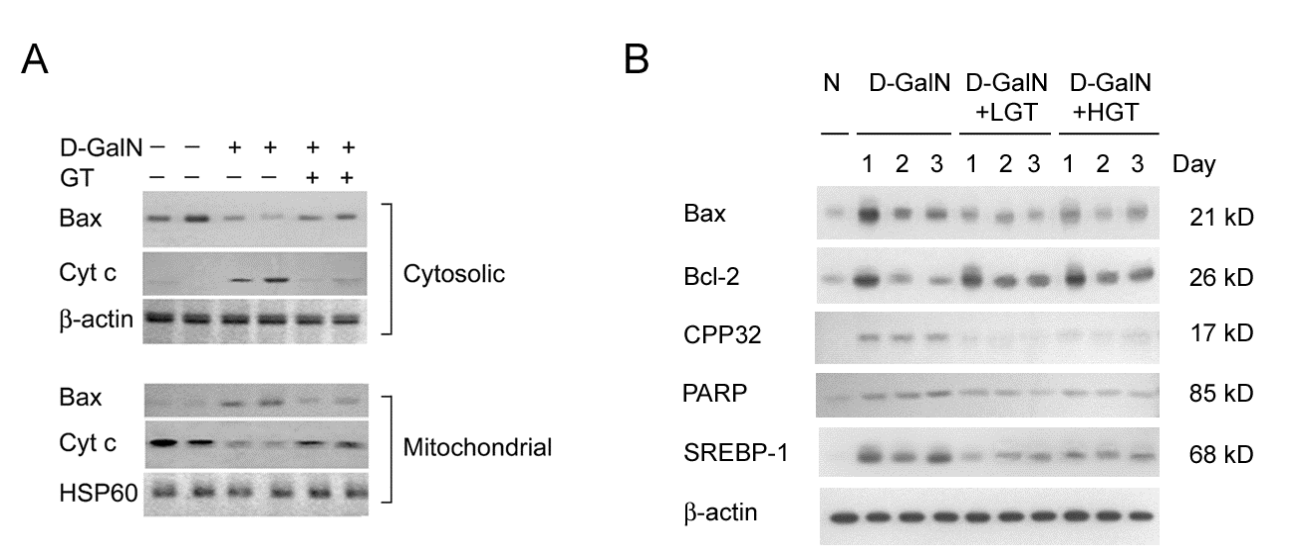
**A: Green tea (GT) extracts inhibit the mitochondrial apoptosis-signaling pathway**. The cytosolic and mitochondrial fractions were prepared at 6 hr after D-GalN from the livers and subjected to western blot analysis for Bax and cytochrome c (Cyt c), respectively. Note that GT treatment decreased the D-GalN-induced cytosolic Bax translocation to mitochondria and accumulation of Cyt c in the cytosol. **B: **Low dose (LGT) and high dose (HGT) of GT decreased D-GalN enhanced proapoptotic Bax/Bcl-2 ratio, CPP32 and PARP expression, and lipogenic enzyme (SREBP-1) in the liver subjected to 1–3 days of D-GalN intoxication.

Similar to the proinflammatory response, the proapoptotic signaling pathway was also enhanced by D-GalN intoxication. D-GalN activated Bax translocation to mitochondria and cytochrome C translocation to cytosol in the livers (Figure 5A). The expression of Bax, Bcl-2, CPP32, and PARP in the total proteins of liver after D-GalN was assessed by immunoblotting with antibodies against Bax, Bcl-2, CPP32, and PARP (Figure 5B). The ratio of Bax/Bcl-2 and the expression of 17 kD (cleaved product) of CPP32 and PARP were all increased in D-GalN livers. Similar to the proapoptotic response, the lipogenic enzyme expression, SREBP-1, was also enhanced after D-GalN treatment. The enhanced proapoptotic and lipogenic signaling could be suppressed by GT pretreatment.

### D-GalN effect on hepatic Kupffer cells, 4-HNE stains and apoptotic cell death

**Figure 6:**
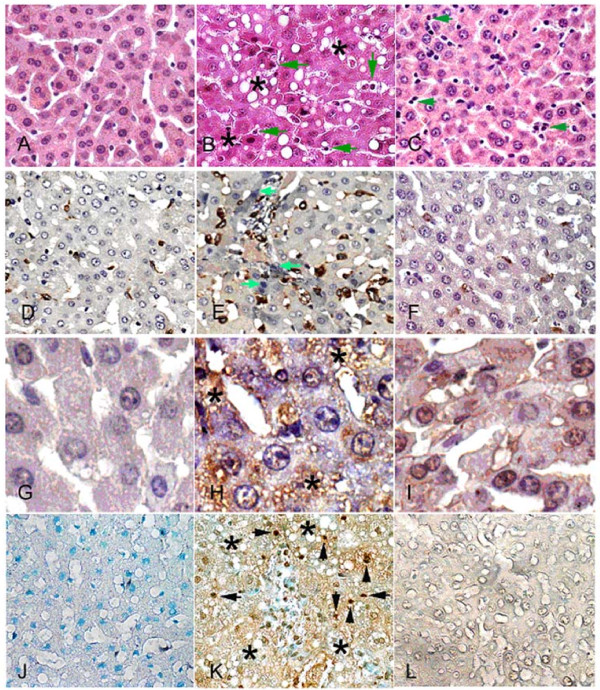
Leukocytic infiltration (indicated by green arrows, B-C), lipogenic accumulation (indicated by stars *, B, H, K), nitroblue tetrazolium (NBT) deposits (blue precipitate indicated by green arrows, E), hepatic Kupffer cell (ED1) stain (brown stain, D-F), 4-hydroxy-2-nonenal (4-HNE) stain (brown color, H), and terminal deoxynucleotidyl transferase-mediated nick-end labeling (TUNEL) stain were used to demonstrate *de novo *production of oxidative stress in the D-GalN treated liver. Oxidative stress indicated by NBT (green arrows) appeared in the Kupffer cells (identified by ED1 brown color) and hepatocytes of rat livers subjected to 24 hr of D-GalN treatment (**E**), but less evident in the control liver (**D**) and the liver with low dose of GT pretreatment (**F**). 4-HNE stain for oxidized protein concomitantly occurred in the D-GalN treated hepatocyte with lipogenic accumulation (**H**), but not appeared in the control (**G**) or GT pretreated livers (**I**). TUNEL stain for apoptotic cell death was absent in the control liver (**J**) and GT treated liver (**L**), but appeared in the liver after 24 hr of D-GalN treatment **(K**). **A-C**, hematoxylin and eosin stain, × 400; **D-F**, NBT+ED1 counterstain, × 400; **G-I, **4-HNE stain, × 600;**J-L**, TUNEL stain, × 400.

Significant lipid accumulation was detected in helatocytes 24–72 hrs after D-GalN treatment (Figure 6). GT pretreatment reduced the appearance of fatty liver (Figure 6C).

A NBT and 4-HNE staining was employted to localize the exact site of ROS generated in the liver. NBT deposits (O_2 _^-. ^generation) did not appear in the livers of control rats (Figure 6D). There was a significant increase in blue formazan deposits in the liver at 24 hr post D-GalN treatment (Figure 6E). The NBT deposits were found in both Kupffer cells (ED1 stain) and hepatocytes.

There was a significant increase in protein oxidation production (brown deposits) in the liver at 24 hr post D-GalN treatment (Figure 6H). The 4-HNE stains were mainly found in the hepatocytes.

ROS oxidized macromolecules and contributed to apoptosis [12,15]. Apoptotic cells were not detected in sections from the control rat liver (Figure 6J). In contrast, apoptotic nuclei were readily be detected in hepatocytes of D-GalN treated livers (Figure 6K). When pretreatment with GT was performed, liver hepatocytes displayed a significant decrease in lipogenic accumulation (Figure 6C), ED-1 (Figure 6F), 4-HNE (Figure 6I), and TUNEL stains (Figure 6L).

### Influence of D-GalN on bile proteomics

For proteomic analysis, 5-μl bile samples from control rats (n = 3), D-GalN rats (n = 5), GT pretreated-D-GalN rats (n = 5) were subjected to 2-DE and proteins visualized by silver staining.

**Figure 7:**
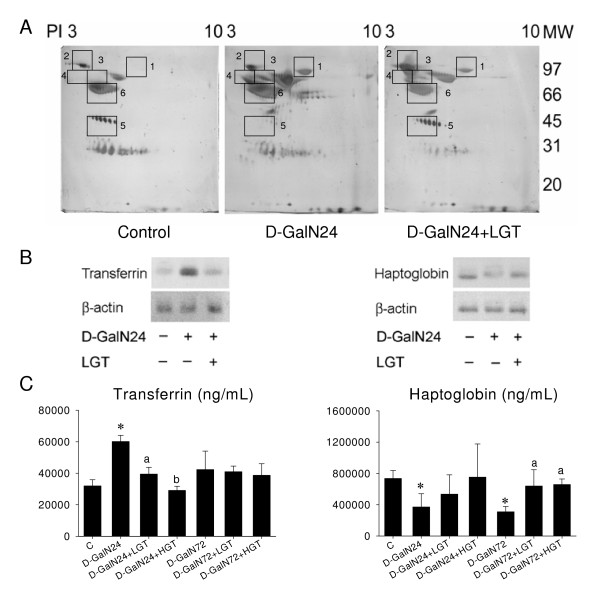
**A: 2-D PAGE electrophotogram of bile**. **B**: Western blotting analysis of transferring and haptoglobin.**C**: ELISA data of bile. Electrophotogram were obtained by silver nitrate staining. The approximate molecular weight (MW) and PI values are as indicated. There were six proteins, spots 1 to 6, with the most striking differences between the control and 24 h after D-GalN treatment (D-GalN24) bile proteome. All of these protein spots (spots 1–6) were subsequently analyzed by MS for protein identification. Whereas transferring (spot 1), polymeric IgA receptor (spot 2), and kallikrein-binding protein (spot 4) expression enhanced at 24 hr and still maintained at high level at 72 hr, the haptoglobin (spot 5) decreased at 24 hr but returned to control levels at 72 hr post D-GalN treatment. Spot 1, transferring; spot 2, polymeric IgA receptor; spot 3, acute phase α-1 protein; spot 4, kallikrein-binding protein; spot 5, haptoglobin; spot 6, Ig α chain. Detailed data is described in Table 1. * P < 0.05 vs. C group; a *P* < 0.05 D-GalN+LGT vs. D-GalN; b *P* < 0.05 D-GalN24+HGT vs. D-GalN24+LGT

Figure 4A shows representative silver stained 2-DE gels of control, D-GalN24 hr, and D-GalN24 hr+GT pretreatment. As shown in Table 1, the protein parameters and the amount of six major proteins identified included transferrin; polymeric immunoglobin A receptor; acute phase α-1 protein; kallikrein binding protein; haptoglobin; and Ig α-chain. After 24 hr post D-GalN treatment, the major biliary proteins, except for hepatoglobin, were increased (Figure 7A). Western blotting (Figure 7B) and ELISA analysis (Figure 7C) confirmed the increased transferrin and decreased haptoglobin level and supported that GT extract pretreatment reduced D-GalN-induced hepatocytes impairment.

**Table 1:** Protein identified in rat bile by 2-D electrophoresis

Spot ID	Accession no.	Protein name	Protein score	Search engine	Molecular weight (Da)	Regulation (D-GalN24/Control)
MASCOT						
1	gi|6175089	Serotransferrin precursor (Transferrin) (Siderophilin) (β-1-metal binding globulin)	52	MASCOT	76314	Increased
2	gi|27151742	Polymeric immunoglobulin receptor; polymeric IgA receptor [Rattus norvegicus]	51	MASCOT	84745	Increased
3	gi|68791	Major acute phase α 1-protein precursor-rat (fragment)	48	MASCOT	46935	
4	gi|92335	Kallikrein-binding protein precursor-rat	59	MASCOT	46490	Increased
5	gi|123513	Haptoglobin precursor	84	MASCOT	38525	Decreased
6	gi|204720	Ig α-chain	61	MASCOT	15594	

MASCOT is a one of the search engine offered by Matrix Science.

### Influence of D-GalN on plasma multiple cytokines

**Figure 8:**
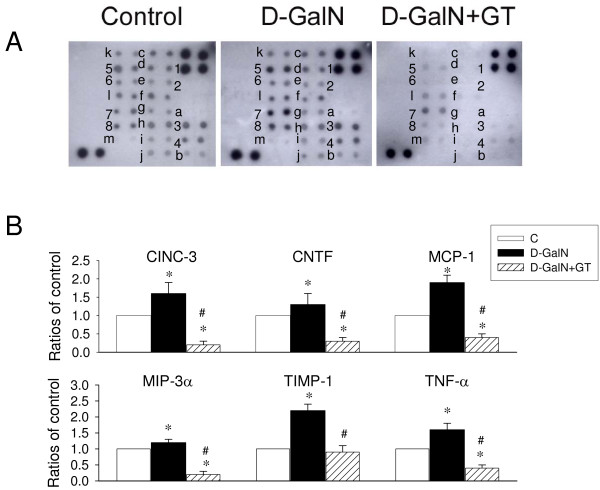
**Determination of multiple cytokines determination by cytokine antibody array in the plasma from control, 24 hr after D-GalN treatment, and 24 hr of after D-GalN and green tea (D-GalN+GT) pretreatment**. The plasma cytokine profiles are displayed in **A**. The mean changes (indicated by ratios of control) of 6 cytokines (n = 3) are displayed in **B**. 1, positive control; 2, negative control; 3, CINC-3; 4, CNTF; 5, MCP-1; 6, MIP-3α; 7, TIMP-1; 8, TNF-α. *P < 0.05, vs. control group. a, cytokine-induced neutrophil chemoattractants-2; b, Fractalkine; c, granulocyte macrophage colony-stimulating factor; d, interferon-γ; e, interleukin-1α; f, interleukin-1β; g, interleukin-4; h, interleukin-6; I, interleukin-10; j, lipopolysaccharide-induced CXC chemokine; k, Leptin; l, β-nerve growth factor; m, vascular endothelial growth factor. # *P *< 0.05, vs. D-GalN group.

Figure 8 depicts multiple cytokines determination by means of cytokine antibody array in the plasma. The mean changes (indicated by ratios of control) of 6 representative cytokines, CINC-3, CNTF, MCP-1, MIP-3α, TIMP-1, and TNF-α were increased after D-GalN intoxication. GT pretreatment significantly depressed the increase of these six plasma cytokine and chemocytokines. Other 13 cytokines indicated from a to m were also measured simultaneously in the plasma. We found that the level of 13 cytokines is not increased after D-GalN treatment when compared to control, but seems to be depressed after GT treatment.

## Discussion

The present study demonstrates that intraperitoneal application of D-GalN induced hepatic sympathetic nerve activation induced hypoxia/hypoperfusion, and increased liver and bile ROS production, hepatocyte apoptosis and proinflammatory cytokines via mitochondrial apoptosis- and proinflammatory cytokine-signaling pathway. GT pretreatment protected against ROS toxicity via enhanced anti-apoptotic mechanism to reduce proinflammatory cytokines and proapoptotic mechanisms in the D-GalN-induced ALI.

Formation of ROS occurs in a variety of forms of liver injury [10-14]. In our study, an early event (within 6 hr) seen after administration of D-GalN is an accumulation of neutrophils and Kupffer cells in the liver sinusoids and an elevation of hepatic and bile ROS. The overproduced ROS may contribute to hepatic apoptotic cell death. Mitochondria are the target and source of ROS [21], which play an important role in physiologic signaling mechanisms and in regulation of apoptotic pathway [22]. Mitochondrial dysfunction disrupts mitochondrial membrane potential (MTP) for ATP synthesis and triggers oxidative and anoxic cell death [23]. The outer mitochondrial voltage-dependent anion conductance (VDAC) channel is involved in cytochrome c release and is regulated by ROS and Bcl-2 family [24,25]. O_2_^-. ^but not H_2_O_2 _induces VDAC-dependent permeabilization of the outer mitochondrial membrane in HepG2 cells [24]. Bcl-2 family is able to regulate the status of MTP; Bax, a channel-forming protein, can open it [26], and Bcl-2 and Bcl-xL are able to stabilize and inhibit its opening [27]. Release of cytochrome c is a proximate trigger for evoking caspase 3 mediated apoptosis [24,25]. Therefore, an increase in ROS and a reduction in Bcl-2/Bax ratio enhance cytochrome c release, and caspase 3 mediated apoptosis [22]. Presently, the decreased ratio of Bcl-2/Bax by D-GalN injury triggers O_2_^-. ^production, Bax translocation to mitochondria, cytochrome c release to cytoplasm, CPP32 activation, and increases PARP fragments initiated apoptosis.

In addition to the apoptotic signaling, the overproduced ROS triggered early cellular signal transduction pathways responsible for the activation of NF-κB and AP-1, resulting in up-regulation of the ICAM-1 gene in the vascular endothelium and tissue accumulation of activated neutrophil accumulation [12]. Likewise, we demonstrated that the damaged liver initiated by D-GalN evoked a burst in the release of ROS that led to early activation of nuclear translocation of the p65 subunit of NF-κB and AP-1, which, in turn, promotes the expression of ICAM-1 protein. This proinflammatory response can be abrogated by GT, which exert antioxidant (scavenging ROS activity) and anti-inflammatory activity (decreasing NF-κB, AP-1, and ICAM-1) on D-GalN enhanced oxidative stress.

In response to oxidative injury or inflammation, acute phase response is initiated [28] and acute phase proteins are predominantly synthesized and secreted by the liver [29]. These acute phase proteins include the polymeric IgA receptor, which is transported from the blood through the transcytotic pathway, transferrin (a hepatocyte-synthesized species involved in uptake by receptor-mediated endocytosis and considered a recycling protein), and haptoglobin, which is delivered from the hepatocyte lysosomal compartment [30,31]. D-GalN increases the biliary level of transferrin in the endosomal recyling compartment and polymeric IgA receptor in the endosomal transcytotic compartment, but decreases that of haptoglobin in lysosomal compartment. These data indicate for the first time that intracellular trafficking of hepatocytes is altered after D-GalN-induced injury.

On the other hand, the acute phase response is also mediated partly by a set of inflammatory mediators such as cytokines and chemokines, which are released by activated macrophages/Kupffer cells or blood monocytes. The liver is the principal target of systemic inflammatory mediators and is the organ responsible supplying the necessary components for tissue damage, for removing harmful agents, and for tissue repair [29]. We have used cytokine arrays in the present study, because RayBio^®^rat cytokine protein arrays are more sensitive and offer higher throughput then the conventional ELISA and Western blotting method [32]. We found that six plasma cytokines and chemokines were elevated after 24 hr of D-GalN intoxication. Two of these (CINC and MIP) are members of the CXC chemokine family, are potent chemotactic factors for neutrophils [33], and contribute to neutrophil recruitment in inflammation [34]. Another affected cytokine (CNTF) is a member of the interleukin-6 (IL-6) superfamily, and is involved in fever induction and a hepatic acute phase protein response [35]. CNTF is removed from the circulation by the liver. Probably as a consequence of hepatocyte binding, CNTF induces acute-phase responses and inflammatory side effects in the liver [36]. A fourth elevated cytokine (MIP-3α) is expressed mostly in the liver and is produced by periportal dendritic cells and/or macrophages after necroinflammatory response, leading to the recruitment of activated T cells into the liver [37]. The plasma concentrations of the final two elevated cytokines (TIMP-1 and TNF-α) are significantly increased in patients with fulminant hepatitis, reflecting severe hepatic inflammation [38]. Inhibition of the nuclear translocation of NF-κB and the reduction of expression of MIP and MCP-1 abrogates hepatitis C virus-infected liver induced leukocyte infiltration [39]. Moreover, we recently demonstrated that GT supplementation markedly attenuates the ROS enhanced proinflammatory NF-κB and AP-1 translocation, ICAM-1 expression, and caspase 3 activity and apoptosis formation in the rat liver [12]. In human study, GT extract supplement palliated plasma HOCl activity more effectively than vitamin C and displayed dose-response effects on reducing inflammation-enhanced H_2_O_2 _and HOCl amounts, atherosclerotic risk factor concentration, C-reactive protein and proinflammatory cytokines expression, and restored paraoxonase 1 activity in high-density lipoprotein [18]. There is no significant dosage effect on ROS level, apoptotic expression in the molecular analysis, and inflammation between low (25 mg) and high GT extract (125 mg) in our study. The level of rat plasma epigallocatechin gallate (10.1 ± 0.3 ng/mL or 13.2 ± 1.2 ng/mL), epicatechin gallate (5.1 ± 0.6 ng/mL or 6.3 ± 0.9 ng/mL), gallocatechin gallate (4.9 ± 0.7 ng/mL or 5.7 ± 0.8 ng/mL) was similar between 25 mg/500 mL and 125 mg/500 mL of GT supplement. We therefore suggest that 25 mg of GT extract may evoke a similar influence as well as 125 mg of GT to reduce D-GalN-induced oxidative stress. Our results confirm the effects of GT and further demonstrated that antioxidant and anti-inflammatory GT activities reduce D-GalN enhanced plasma proinflammatory cytokines.

D-GalN or alcohol is well known substances which produce toxic liver damage in animal experiments. Of special significance is the toxic fatty infiltration in the live parenchyma of these models [2,6]. Oxidative stress originating from overexpression of an active form of SREBP-1 and increased intracellular levels of fatty acids has also been implicated as a cause of hepatocellular injury in steatosis [40]. Our data shows that D-GalN induced fatty acid production in the liver, possibly via the elevation of SREBP-1 protein expression. Immunocytochemical analysis revealed the concomitant accumulation of 4-HNE and lipogenic products in D-GalN treated liver. Amelioration of hepatic steatosis caused by SREBP-1 gene disruption in ob/ob mice lowers triglyceride content [41]. A diet enriched in fish oil can downregulate lipogenesis by decreasing liver SREBP-1, suppressing endogenous PPARαactivation, and increasing antioxidant gene expression, thereby protecting against ROS excess [42]. Presently, we demonstrate that GT markedly decreases the mature form of SREBP-1 protein and reduces the expression of lipid accumulation in the D-GalN treated rats. GT ameliorates hepatic steatosis, presumably through downregulation of oxidative stress enhanced SREBP-1.

Using antioxidants such as vitamins E and C or ROS scavengers such as catalase or superoxide dismutase prevented acute hepatocellular damage [42]. Previously, the apoptotic protection of epigallo-catechin gallate, a major component of catechins, was via the suppression of the elevated caspase-3 in the cytoplasm [12]. The present study demonstrates low and high doses of GT offered direct and indirect protection in reduction of plasma ALT and cytokine levels, improved liver histology, preserved antioxidant and anti-apoptotic protein content, and decreased ROS production in the liver and bile from animals receiving GT compared with those receiving water. The protection may be attributed to the antioxidant, anti-apoptotic, and anti-inflammatory activity of GT in the liver cells and in the interstitial space.

In conclusion, D-GalN induces ALI via mitochondrial apoptosis- and proinflammatory cytokine-signaling pathways, subsequently leading to increases in ROS production, hepatocyte apoptotsis, and plasma multiple cytokines and chemokines. GT can counteract D-GalN-induced mitochondrial apoptosis- and proinflammatory cytokine-signaling pathways by the possible mechanisms of a direct scavenging ROS activity and an upregulation of antioxidant defense mechanisms.

## Abbreviations

ALI: acute liver injury; ALT: alanine aminotransferase; AP-1: activator protein-1; Bcl-2/Bax: apoptotic Bcl-2 family proteins; CINC-3: cytokine-induced neutrophil chemoattractant; CNTF: ciliary neurotrophic factor; CPP32: caspase 3; 2-DE: 2-dimensional electrophoresis; D-GalN: D-galactosamine; EMSA: electrophoretic-mobility shift assay; ED1: hepatic Kupffer cell; GT: green tea; 4-HNE: 4-hydroxy-2-nonenal; HPLC: high performance liquid chromatography; HSP60: heat shock protein 60; ICAM-1: intercellular adhesion molecule-1; MCLA: 2-Methyl-6-(4-methoxyphenyl)-3,7-dihydroimidazo- [1,2-a]-pyrazin-3-one-hydrochloride (MCLA); MALDI: matrix-assisted laser desorption/ionization; MALDI-TOF MS: MALDI time-of-flight mass spectrometry; MCP-1: monocyte chemoattractant protein-1; MIP-3α: macrophage inflammatory protein 3 alpha; MTP: mitochondrial membrane potential; NBT: nitroblue tetrazolium; NF-κB: nuclear factor-kappa B; PARP: poly-(ADP-ribose)-polymerase; PVBF: portal venous blood flow; PVP: portal venous pressure; ROS: reactive oxygen species; SREBP-1: sterol regulatory element binding protein-1; TIMP-1: tissue inhibitor of metalloproteinases-1; TNF-α: tumor necrosis factor-alpha; TUNEL: terminal deoxynucleotidyl transferase-mediated nick-end labeling; VDAC: outer mitochondrial voltage-dependent anion conductance.

## Competing interests

The authors declare that they have no competing interests.

## Authors' contributions

BRL, YCL, CTC and CFC conceived the hypothesis, contributed to the design and conduct of the study, conducted the statistical analyses, drafted the manuscript and critically revised manuscript. WCC, HSL, and HMC provided important comments and excellent techniques in the paper. All authors read and approved the final manuscript.
